# Pyogenic Flexor Tenosynovitis as a Rare Complication of Dyshidrotic Eczema

**DOI:** 10.5811/cpcem.2020.1.45414

**Published:** 2020-03-27

**Authors:** Waroot S. Nimjareansuk, Michael Rosselli

**Affiliations:** Mount Sinai Medical Center, Department of Emergency Medicine, Miami Beach, Florida

**Keywords:** flexor tenosynovitis, pyogenic, eczema, dyshidrotic, ultrasound

## Abstract

**Introduction:**

Pyogenic flexor tenosynovitis is an unusual complication of dyshidrotic eczema. The diagnosis has traditionally been made by Kanavel’s signs. Point-of-care ultrasound can be a useful adjunct in the diagnosis of this surgical emergency.

**Case Report:**

We report the case of a 23-year-old male who presented with right middle finger pain and swelling and an overlying eczematous rash. The use of point-of-care ultrasound was performed to aid in the diagnosis of pyogenic flexor tenosynovitis. An incision and drainage was performed with deep wound cultures positive for *Staphylococcus aureus*.

**Discussion:**

The presentation of pyogenic flexor tenosynovitis with underlying concomitant dermatological disease can complicate this challenging diagnosis. Point-of-care ultrasound can be an effective adjunct in revealing pyogenic flexor tenosynovitis rather than relying solely on the classical Kanavel’s signs, leading to earlier treatment.

**Conclusion:**

Our case demonstrates that point-of-care ultrasound can be a rapid and effective tool for the diagnosis of pyogenic flexor tenosynovitis in the setting of superimposed dermatological diseases.

## INTRODUCTION

Pyogenic flexor tenosynovitis (PFT) is an acute infection of the synovial flexor tendon sheath often presenting first to the emergency department (ED). This aggressive, closed-space infection is classically diagnosed in patients presenting with the four Kanavel’s signs: uniform swelling; tenderness at the flexor tendon sheath; the digit held in flexion; and pain on passive extension.^1^ The natural history of PFT usually presents two to five days after a penetrating injury and rarely by hematogenous spread.^2^ Bacterial introduction into the skin is a common occurrence in those with predisposing dermatologic diseases as a result of self-induced skin breakdown or skin microbiome alterations owing to an imbalance of staphylococci over streptococcus.^3^ However, the large majority of cases result in cellulitis and not in PFT. The diagnosis of PFT is challenging but one that must be made at the patient’s first encounter as it is a surgical emergency. In conjunction with the clinical exam findings, point-of-care ultrasound POCUS) can assist with the diagnosis of PFT and, therefore, avoid loss of hand function or amputation in severe cases.^4^

## CASE REPORT

A 23-year-old right-handed male presented to the ED with right middle finger pain and swelling throughout the day. The swelling rapidly progressed over four hours predominately at the palmar aspect of the middle finger. The patient reported working as a chef and wore gloves on a daily basis. He denied trauma, fever, chills, sore throat, joint pain, penile discharge, or genital sores. He had a history of eczema mainly affecting the bilateral palmar hands and had allergies to dust, pollen, and cat dander. The patient reported using Cetaphil cream and took loratadine and diphenhydramine often. He denied any allergies to medication and had no surgeries in the past. The patient had been sexually active with one female partner for the prior six years and denied any history of sexually transmitted infections. He smoked half a pack of cigarettes per day for three years but quit one month prior. He also drank alcohol twice per month and smoked marijuana once per month.

**Image 1:**
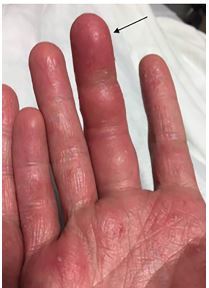
Palmar aspect of the patient’s right hand. There is erythema and swelling (black arrow) of the right middle finger held in slight flexion. An associated scaly eczematous skin eruption is shown.

**Image 2:**
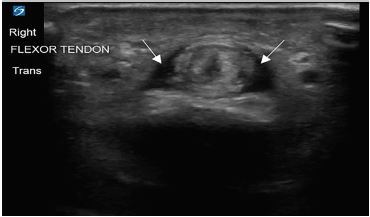
Transverse ultrasound view of the right middle finger. The image displays anechoic fluid (white arrows) surrounding the flexor tendon within the tendon sheath.

**Image 3:**
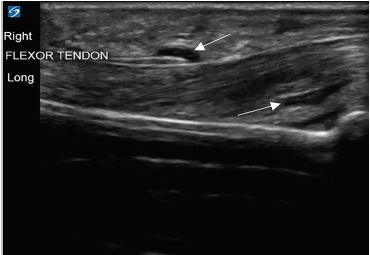
Longitudinal ultrasound view of the right middle finger. The image displays the striated flexor tendon with anechoic fluid (white arrows) above and below the tendon.

His vitals in the ED were oral temperature 98.4 degrees Fahrenheit, heart rate of 77 beats per minute, blood pressure 128/76 millimeters of mercury, respiratory rate of 17 breaths per minute, and oxygen saturation of 99% on room air. He was well appearing and in no acute distress. His right middle finger was held in slight flexion with fusiform swelling and tenderness at the flexor surface (Image 1). He had pain with passive extension of his finger but sensation was intact. He also had a scaly erythematous skin eruption with desquamation and excoriations of his bilateral palms with several one-millimeter pustules. Laboratory testing revealed a leukocytosis of 12.69 × 10^3^ cells per liter (L) (reference range: 4.8–10.8 × 10^3^ cells/L) with left shift and plain film radiographs of the right hand and fingers were unremarkable. An ED POCUS of the right middle finger was performed showing anechoic fluid within the flexor tendon sheath (Images 2 and 3).

**CPC-EM Capsule**

What do we already know about this clinical entity?

Pyogenic flexor tenosynovitis (PFT) has traditionally been diagnosed using Kanavel’s signs and requires emergent surgical intervention.

What makes this presentation of disease reportable?

PFT is a vital but rare diagnosis in the setting of concomitant overlying dermatological diseases.

What is the major learning point?

Point-of-care ultrasound (POCUS) can be used as an adjunct to diagnose PFT in the setting of an uncertain clinical picture.

How might this improve emergency medicine practice?

Emergency physicians can use clinical findings with the aid of POCUS to solidify the diagnosis of PFT.

Based on clinical exam and ultrasound findings, the patient was diagnosed with flexor tenosynovitis of the right middle finger and started on vancomycin and ceftriaxone intravenously. The patient was admitted to the hospital with hand surgery consultation. He subsequently underwent an incision and drainage. Deep wound cultures were positive for *Staphylococcus aureus* with blood cultures producing no growth. Subsequent gonorrhea, syphilis, and human immunodeficiency virus-1/2 testing was negative. Dermatology was consulted and the patient was diagnosed with atopic dermatitis with dyshidrotic eczema. Dermatology recommended that the patient be treated with triamcinolone 0.1% ointment once a day, clobetasol 0.05% ointment once a day, and hydroxyzine 25 milligrams at bedtime as needed. The patient improved and was discharged home on hospital day three status post incision and drainage with amoxicillin-clavulanic acid for five days and outpatient follow-up with hand surgery.

## DISCUSSION

Flexor tenosynovitis is a time-sensitive diagnosis that can result in severe morbidity due to complications such as adhesions, deep space infection, tendon rupture, or amputation.^2^,^4^,^5^ Although traditionally diagnosed by the classic Kanavel’s signs, there is no published study validating these signs. Dailiana et al showed that only 54% of patients had all four signs in a retrospective review.^6^ Two studies have evaluated the individual signs among patients with PFT. Pang et al showed that fusiform swelling was the most common sign in patients diagnosed with PFT and that pain along the flexor tendon sheath was a late sign.^7^ In addition, Neviaer and Gunther proposed that the earliest Kanavel’s sign was pain on extension and also proposed that the inability to flex the finger entirely to the palm was an additional sign of PFT.^8^

To further complicate a challenging clinical diagnosis, most cases of PFT are caused by direct inoculation from trauma to the finger and may appear minor or even innocuous from a scrape or a scratch.^9^ This was likely the case in our patient with dyshidrotic eczema. Secondary bacterial infection of dyshidrotic eczema resulting in cellulitis is not uncommon.^10^ Thus, POCUS in the ED can be a useful adjunct to elucidate the diagnosis of PFT vs cellulitis.

The normal flexor tendon sheath does not have an appreciable amount of fluid when visualized on ultrasound as the parietal and visceral layers of the sheath form a sealed synovium.^11^,^12^ Hypoechoic or anechoic fluid within the flexor tendon sheath has been shown to correlate with purulent discharge upon surgical intervention.^13^,^14^ A study published by Jardin et al showed ultrasound findings of peritendinous effusion, and thickened synovial sheath had a sensitivity of 94%, specificity of 74%, and negative predictive value of 96.7%.^15^ Therefore, the presence of fluid within the flexor tendon sheath can aid in the diagnosis of PFT.

## CONCLUSION

Pyogenic flexor tenosynovitis may not present with all the typical Kanavel’s signs. To make the detection of the disease even more challenging, overlying dermatologic diseases may mask this diagnosis. Performing a POCUS in the ED is an effective and timely adjunct to support the diagnosis of PFT, leading to earlier treatment with antibiotics and/or surgical intervention.
